# Impact of the COVID-19 pandemic on medical students of clinical clerkship in South Korea: A qualitative study exploring medical students’ experiences

**DOI:** 10.12669/pjms.38.3.5031

**Published:** 2022

**Authors:** Kwi Hwa Park, So Jung Yune, Min Kyu Jung, Yura Kim, Geon Ho Lee, Seong Yong Kim

**Affiliations:** 1 Kwi Hwa Park, Department of Medical Education, Gachon University College of Medicine, Namdong-gu, Incheon, 21565, Korea (South); 2 So Jung Yune, Department of Medical Education, Pusan National University College of Medicine, Mulgeum-eup, Yangsan, 50612, Korea (South); 3 Min Kyu Jung, Department of Internal Medicine, Kyungpook National University School of Medicine, Jung-gu, Daegu, 41944, KOREA (South); 4 Yura Kim,Department of Medical Humanities, Collage of Medicine, Yeungnam University, Nam-gu, Daegu, 42415, KOREA (South); 5 Geon Ho Lee, Department of Family Medicine, School of Medicine, Daegu Catholic University, Nam-gu, Daegu, 42472, KOREA (South); 6 Seong Yong Kim, Department of Medical Education, Collage of Medicine, Yeungnam University, Nam-gu, Daegu, 42415, KOREA (South)

**Keywords:** Phenomenology, Qualitative study, Medical students, COVID-19 pandemic

## Abstract

**Background and Objective:**

In 2020, during the coronavirus disease (COVID-19) pandemic, medical students were placed in a learning environment that exposed them to unsafe clinical settings. In this study, using a phenomenological approach, we analyze the experiences of fourth-year students in the Daegu area of South Korea, a region that experienced a high concentration of COVID-19 infections.

**Methods:**

The essays of 80 students from four medical schools who agreed to participate in the study were utilized in the final data analysis. The data were analyzed using the proposed phenomenological analysis.

**Results:**

Forty-seven condensed meaning units, twelve subthemes, and three essential themes were identified. The main theme includes the following: 1) confusion and stress due to sudden changes in the learning situation 2) learned the medical professionalism of physicians 3) reflection and internal change regarding what it means to be a physician.

**Conclusions:**

The COVID-19 pandemic had a significant impact on students who participated in clinical clerkships. This study can provide baseline data for planning educational strategies and establishing a support system for students in response to the changes that they may experience in the event of the reoccurrence of a novel infectious disease in the future.

## INTRODUCTION

The world faced an unprecedented pandemic in 2020 with the rapid spread of the novel coronavirus disease (COVID-19). In March 2020, the Daegu area of South Korea was declared a special disaster zone, as the region experienced an explosive growth of confirmed COVID-19 cases. Accordingly, the four medical schools in this area converted their in-person classes to virtual classes offered online, except for their clinical clerkship programs. At that time, the schools monitored the trend of COVID-19 cases and postponed the start of clinical clerkships, which primarily pertained to third-and fourth-year students, for over a month. However, medical students in South Korea must undergo at least 52 clinical trainings, according to the Korean Institute of Medical Education and Evaluation; students began their clinical clerkships in April 2020 amid feelings of concern and anxiety.

When such an epidemic occurs, the medical community’s primary concern is managing infectious diseases and improving the disease management systems.^1^ Health care workers were also at risk, with health care workers accounting for 15 to 18% of the total infected population, and in some cases up to 20%.^2^ This dangerous situation was the same for students taking classes in hospitals. Medical schools were not only interested in their patients’ safety but also developed guidelines to prevent student infection and ensure their safety.

The occurrence of such outbreaks is also a source of psychological stress among medical students. Most medical students experienced great stress and a certain degree of anxiety. 3-4 As a result, emphasis was placed on the necessity of educating medical students on infection prevention, which included knowledge of and attitudes regarding infectious diseases and infection prevention measures.^5^

While many university hospitals altered or suspended their clinical clerkship schedules during the last MERS outbreak, the confusion and changes experienced during the current COVID-19 pandemic have been unprecedented. This alone place medical students in a new and challenging situation. Moreover, students’ direct and indirect experiences will differ from those before the pandemic. However, studies have not analyzed medical students’ experiences during this COVID-19 pandemic, the meaning of these experiences, and their impact on students.

Therefore, the purpose of this study was to analyze the experiences and personal changes’ meaning of medical students who received clinical training at medical schools in the Daegu area of South Korea using the phenomenological method.

## METHODS

**Fig.1:**
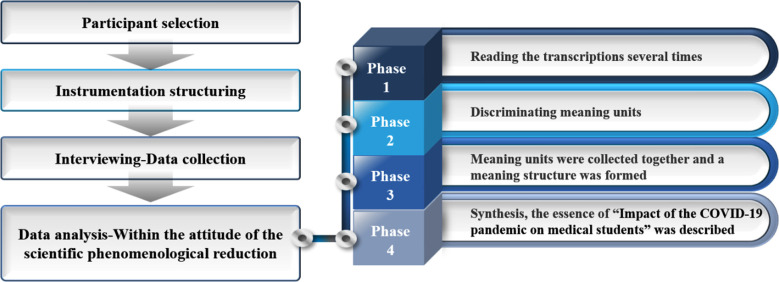
Procedure for phenomenological approach.

The phenomenological study is an inductive and descriptive research method that aims to determine the meaning behind lived experiences/phenomena by analyzing their descriptions.^5^ The procedure for phenomenological approach is shown in Fig.1.

The subjects in this study consisted of a convenience sample of fourth-year medical students attending four medical schools in the Daegu area of South Korea, which had the highest distribution of confirmed COVID-19 cases nationwide at 63.7% as of April 30, 2020. The students participated in clinical clerkship programs and were scheduled to take the Korean Medical Licensing Examination (KMLE) as soon-to-be graduates in 2020. For the final data analysis, this study utilized the questionnaires of 80 students who agreed to participate in the study after seeing a recruitment advertisement for research participants (Table-I).

**Table-I:** Research subjects.

	School	Male	Female	Total
Medical School	A	15(62.5)	9(37.5)	24(100.0)
	B	9(64.3)	5(35.7)	14(100.0)
	C	12(63.2)	7(36.8)	19(100.0)
	D	15(65.2)	8(34.8)	23(100.0)
	Total	51(63.8)	29(36.2)	80(100.0)
Age	Mean age 25.46 years (22~35)			

Data were collected between May and July of 2020. After obtaining permission from each medical school to conduct a survey, emails were sent to students with the address of an online survey site that contained a recruitment advertisement for research participants and a consent Form. The questionnaire was composed of semi-structured open-ended questions, and the resulting responses were saved in a file format. The questionnaire items were verified by two medical school professors and two qualitative researchers. Students were asked to respond to each question by describing their experiences in detail. They were also asked to respond to the question, “Why did you think so?” “How did you react?” and “How did you feel at the time?” realistically. Moreover, they were asked to compose their essays in a way that effectively demonstrated the COVID-19 situation.

According to Giorgi’s phenomenological analysis process^6^, the collected data were analyzed to understand the experiences of medical students amid the COVID-19 pandemic. Following Lincoln and Guba’s evaluative criteria regarding the rigor of qualitative research^7^, this study aimed to ensure the truth value, applicability, consistency, and neutrality of the research. The Daegu Joint Institutional Review Board reviewed the present study (IRB No. DGIRB 2020-04-001).

## RESULTS

**Fig.2:**
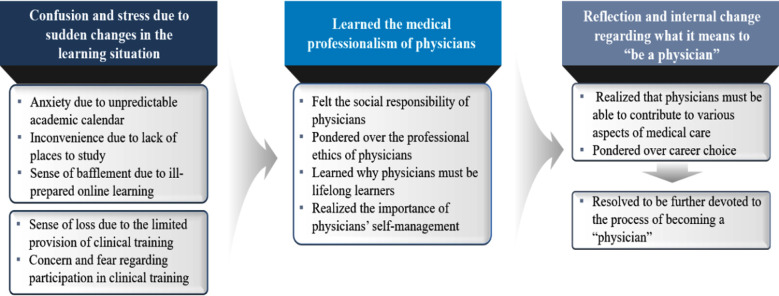
Subthemes and essential themes.

Forty-seven condensed meaning units were derived according to Giorgi’s method of analysis.^6^ Twelve subthemes and three essential themes were then obtained by grouping the condensed meaning units into responses based on their common content (Fig.2).

### Confusion and stress due to sudden changes in the learning situation:

### Anxiety due to unpredictable academic calendar

Because of the pandemic, it became difficult for schools to operate according to their planned academic calendars. No one could guarantee when classes would resume. As the participants in this study were fourth-year medical students who had to complete the fourth-year curriculum and take the KMLE, they were left with material to study even when everything came to a standstill. Moreover, when classes resumed, they had to deal with a tight class schedule, in addition to exams.

*It had a huge negative impact on fourth-year students who focused more on their clinical training than on school classes. We were not able to perform our clinical training for the month of March, so we had to continue our training until July and August to meet the academic schedule. (C school, Female)*

### Inconveniences due to the lack of places to study

Students could no longer use the campus learning environment to study. Students living with their families had difficulty concentrating on their studies, and those living on their own had to remain in their rooms for several months. When students lost the use of places where they usually spent most of their day, it was a great inconvenience and caused loneliness.

*When campus facilities were closed, the use of reading rooms and libraries was limited. More students are going to places, such as cafes, because they cannot use the campus library’s reading rooms, but would it not be more dangerous to be exposed to many random people at the I? (School A, Female)*

### Sense of bafflement due to ill-prepared online learning

It was difficult for students and professors to adapt to online learning when they had not been prepared for it. Students experienced both convenience and frustration when listening to online lectures. As many professors had never prepared online lectures before, they also felt like they had become novice professors once more.

*Most of our classes were replaced by live or recorded online lectures, but honestly…While I realize that the professors were also having difficulty with the unfamiliar method, they were very amateurish (D school, Male)*

### Sense of loss due to the limited provision of clinical training

The most significant change brought about by the COVID-19 pandemic was the change to an un-tact (non-face-to-face) society. To prevent person-to-person contact, hospitals limited contact between patients and healthcare providers and limited the movement paths within hospitals. It was no longer possible for students to attend seminars or conferences with professors or have private talks outside of class where students could freely ask questions and learn. Students gradually became more passive with their clinical training and feared meeting patients.

*I think I was a lot more cautious than I had been last year whenever I interacted with a patient. As we were trying not to put each other at risk, it was difficult to readily carry out preliminary examinations for outpatients, take the history of patients for case presentations, perform physical examinations, and more (C school, Female)*.

### Concern and fear regarding participation in clinical training

Clinical clerkship students stand on the border between healthcare providers and the public. Students encounter outsiders in their daily lives while simultaneously meeting patients at the hospital. Therefore, students were anxious that they might spread COVID-19 to others and worried that they might become infected by patients at the hospital. Students gave up much of their daily lives to follow preventive measures against COVID-19. Therefore, when these students saw colleagues who were not complying with such measures, they experienced resentment and even dislike. Students were worried that they might become ill and feared that they may affect their family, colleagues, and patients.

*When we are at the hospital, we are neither outsiders nor insiders. That is why I would sometimes think we posed a greater threat as potential hosts of pathogens than did health care workers who stayed at the hospital for a long time or patients who visited the hospital for short periods of time (D school, Female*). ))))

### Learned the medical professionalism of physicians:

### Felt the social responsibility of physicians

The sweat and tear-stained faces of professors and senior colleagues in protective suits made students realize that being a physician signified more than honor and affluence and that one could not be a physician without having a sense of duty and the resolve and willingness to risk ‘one’s life. Students were able to feel the weight of the social responsibility placed on physicians.

*When I saw physicians working in the field and physicians who had traveled far to volunteer, I thought it was incredible. I admired them even more because when I thought about whether I would be able to make the same decision as them in the future, I could not come up with a quick answer (A school, Female)*

### Pondered over the professional ethics of physicians

Many physicians were gathered in the Daegu area to treat patients with COVID-19. However, some physicians refused to see the COVID-19 patients. Students observing the latter thought deeply about whether the physicians’ refusal to treat patients was valid. While some students criticized these physicians and said their cowardly attitude went against their professional ethics, others argued that sacrifices made by physicians should not be taken for granted.

*In March, when there was an outbreak of COVID-19 cases in Daegu, I saw some physicians refusing to see suspected patients with symptoms such as fever when they came to their hospital, and I thought about whether such actions could be justified. I wondered if there could have been another option instead of refusing them (D school, Male)*.

### Learned why physicians must be lifelong learners

Students saw how frightening it was to treat someone without having the necessary knowledge. They realized that physicians must be knowledgeable and that it is their duty to provide the public with accurate information. Students understood that the professional ethics of physicians necessitated lifelong learning.

*As COVID-19 was a novel virus that even physicians had not heard of or learned about, it was difficult to diagnose and treat. More so than anyone, physicians felt that it was necessary to quickly update and apply information regarding global cases and treatment guidelines to provide the best care (A school, Male)*.

### Realized the importance of physicians’ self-management regarding risk of infection and transmission

If a physician contracts COVID-19, the risk of becoming a super spreader increases two-fold. Clinical clerkship students saw how meticulously physicians managed their lives to prevent the risk of infection. As no one is free from this fear, the following students expressed their admiration for senior colleagues who were meticulous in managing their lives.

*As the following preventive measures have become very important at present, I think becoming a “role model” should be the top priority of physicians. Physicians must pay greater attention to preventive measures than others, and take the lead in social distancing. Most physicians practiced these measures themselves (School A, male)*.

**Reflection and internal change regarding what mean to be a physician.**

### Understood the diverse roles of physicians beyond that of treating patients:

Experiencing the COVID-19 pandemic made students-who had thought that a physician’s work was limited to treating patients-realize that the scope of a physician’s responsibility is broad. Being a physician includes helping establish legislation or systems, promoting disease prevention and public education, acting as a spokesperson for the field of health care, and taking the lead as a role model for the public.

*I came to think that a physician’s role not only included diagnosing and treating patients with COVID-19. I felt that physicians must also oversee epidemiological investigations at the Disease Control and Prevention Agency, present related policies, and manage life management centers (B school, Male)*

### Pondered over career choice

Everyone had a horrifying experience in the Daegu area, which at one point had the largest number of confirmed COVID-19 cases in South Korea. Students saw physicians treating patients under such circumstances, even at the risk of becoming infected, and students who felt aggrieved that these physicians were not being treated properly expressed their doubts about a physician’s career. The COVID-19 pandemic served as an opportunity for students to think about the profession of a physician once again.

*Due to the nature of Korea’s college entrance exams, many students who entered medical schools simply looked forward to a prosperous future. However, a physician’s duties were emphasized even more with the current situation, and when I saw that hardworking physicians were being exploited and forced to accept “passion pay” in addition to being used politically without being treated properly, I began to have doubts about my career path (C school, Male)*

### Resolved to be further devoted to the process of “becoming a physician”

When students saw their professors or senior colleagues amid the COVID-19 pandemic, they reflected on how they had studied to receive good grades and avoided being held back at their medical school. Moreover, students said they became committed to studying in-depth and participating more actively in their clinical training. They pledged to devote more effort to learning.

I was thinking that I would also like to become a great healthcare provider who could deal with emergency situations. That is why I am planning to work harder during my clinical training (D school, Male).

## DISCUSSION

Students experienced confusion and stress because of the sudden, rapid changes in the medical learning environment, which they were not prepared for, due to the COVID-19 pandemic. Students felt stressed and burdened with academic pressure because of the unpredictable academic calendar, such as the fact that exams were being delayed and courses did not follow the scheduled curriculum. Moreover, students could no longer use campus learning environments. Students were greatly inconvenienced and experienced loneliness when they lost the use of places where they had spent most of their days. These results were consistent with the findings of other studies, which stated that the COVID-19 pandemic impacted the medical curriculum and the well-being of students, and did not positively affect their lifestyles.^8^ Uncertainty and ambiguity were significant factors that psychologically affected students.^9^

When online learning has not been well prepared, it causes learners and instructors to feel baffled. In four universities, both learners and instructors had an inadequately prepared online learning experience, resulting in many trials and errors. The switch to online learning was inevitable during the COVID-19 pandemic.^10^ However, as online classes are conducted entirely with technology, different instructional design strategies are needed, in contrast to those used for face-to-face classes.^11^ Furthermore, the gap between instructors and learners’ digital literacy can also add to the confusion.^12^ As online teaching methods are expected to become established as a new paradigm even after the Covid-19 period^13^, efforts must be made at the university level, such as by building a learning environment that is suitable for this change and by developing programs to strengthen the digital competence of instructors and learners.

Students experienced a sense of loss because they felt they were not being provided with adequate learning opportunities even though they had begun their clinical clerkships after much difficulty. However, it was reported that medical students who perceived themselves as preliminary health care providers were less concerned about becoming infected during their clinical training and had positive attitudes regarding their participation in clinical clerkships.^14^ Above all else, as clinical training cannot be substituted with online classes, it is even more necessary to prepare specific codes of conduct and guidelines to promote the safety of those involved. In addition, this suggests the need to develop diverse instructional strategies so that students can receive their clinical training in safer ways, such as by participating in telemedicine, virtual rounds and conferences, clinical performance-based role play, and treating virtual patients.^15^

Students learned the medical professionalism of physicians because of the COVID-19 pandemic. For medical students, it is important to have role models that help them form their professional attitudes. When physicians showed students that they were doing their utmost as health care providers on the front lines of treating COVID-19 patients, and how meticulously they were managing their lives amid the risk of infection and infecting others, they provided great role modeling for the students. Meanwhile, students faced a dilemma of professional ethics and experienced a conflict in the process when they saw physicians refusing to see patients who were suspected of having COVID-19. Such ethical experiences provided students with the opportunity to reflect on the ethical issue of whether physicians must fulfill their professional obligations regardless of risks. Students became aware of the social responsibility placed upon the profession of physicians and learned that physicians must be lifelong learners. Physicians must also be able to deal with sudden changes, assess changes, and apply them in a way that improves patient treatment.^16^ Continuing professional development in health care has become even more important due to the increase in various unpredictable diseases.

### Limitations of the study

As the present study only focused on medical students who were receiving clinical training in the Daegu area of South Korea, which had the highest distribution of confirmed COVID-19 cases during the onset of the pandemic, there are limitations to the transferability and generalizability of this study’s findings. Furthermore, because of the South Korean government’s COVID-19 preventive guidelines, we could only collect written data through reflective essays. In future studies, interviews must be conducted to collect additional data and compensate for these limitations.

## CONCLUSION

Medical students displayed a mature attitude in their attempts to improve their shortcomings and reflect on their present selves regarding how devoted they were to the process of “becoming a physician” and how prepared they are. This self-reflection could help medical students, who are also preliminary health care providers, establish their sense of identity. By experiencing the COVID-19 pandemic, students recognized that a physician’s responsibilities must extend beyond their basic role of treating patients.

### Authors Contributions:

**KHP:** Original Draft, Methodology, Formal analysis.

**SJY:** Methodology, Formal analysis, Data Curation, Visualization.

**MKJ:** Investigation, Formal analysis.

**YRK:** Investigation, Formal analysis, Project administration.

**GHL:** Conceptualization, Supervision, Writing-Review & Editing.

**SYK:** Writing-Review & Editing, Supervision, Funding acquisition.
